# Ethanolic Extract of Melia Fructus Has Anti-influenza A Virus Activity by Affecting Viral Entry and Viral RNA Polymerase

**DOI:** 10.3389/fmicb.2017.00476

**Published:** 2017-03-28

**Authors:** Young-Hee Jin, Jang-Gi Choi, Won-Kyung Cho, Jin Yeul Ma

**Affiliations:** Korean Medicine Application Center, Korea Institute of Oriental MedicineDaegu, South Korea

**Keywords:** Meliae Fructus, influenza virus, A/PR/8/34, H3N2, MDCK, viral entry, PA

## Abstract

Meliae Fructus (MF) is the dried ripe fruit of *Melia toosendan* Siebold et Zuccarini, Meliaceae family. MF is widely used in traditional medicine to treat inflammation and helminthic infection and has anti-bacterial, anti-oxidant, anti-cancer, anti-inflammatory, and analgesic activities. However, potential anti-influenza properties of MF have yet to be investigated. We determined whether an ethanolic extract of MF (EMF) has anti-viral activity via an EMF pre-, co-, and post-treatment assay, using the Influenza A/PR/8/34 and H3N2 virus on Madin-Darby canine kidney (MDCK) cells. The EMF had anti-influenza virus activity in pre- and co-treated cells in a dose-dependent manner, but not in post-treated cell. EMF inhibited the activity of hemagglutinin (HA) and neuraminidase (NA) of influenza virus. EMF inhibited viral HA, nucleoprotein (NP), matrix protein 2 (M2), non-structural protein 1 (NS1), polymerase acidic protein (PA), polymerase basic protein 1 (PB1), and polymerase basic protein 2 (PB2) mRNA synthesis at 5 h post infection (hpi), however, the levels of PA, PB1, and PB2 mRNA were increased in pre- and co-EMF treated cells compared with control virus-infected and EMF post-treated cells at 18 hpi. The level of M2 protein expression was also decreased upon pre- and co-treatment with EMF. The PA protein was accumulated and localized in not only the nucleus but also the cytoplasm of virus-infected MDCK cells at 18 hpi. Pre-EMF treatment inhibited the expression of pAKT, which is induced by influenza virus infection, at the stage of virus entry. We also found that treatment of EMF up-regulated the antiviral protein Mx1, which may play a partial role in inhibiting influenza virus infection in pre- and co-EMF treated MDCK cells. In summary, these results strongly suggested that an ethanolic extract of Meliae Fructus inhibited influenza A virus infection by affecting viral entry, PA proteins of the RNA polymerase complex, and Mx1 induction and may be a potential and novel anti-influenza agent.

## Introduction

Influenza A virus causes seasonal and endemic infection, periodic and unpredictable pandemics, high morbidity and high mortality to human and several kinds of animals such as birds and pigs (Taubenberger and Morens, 2008). The Spanish Influenza virus (H1N1) killed around 50 million people in 1918 and was the worst human outbreak (Taubenberger and Morens, 2006). Other pandemics occurred in 1957 (H2N2), 1968 (H3N2), 1977 (H1N1), 1997 (avian H5N1), 2009 (swine H1N1), and 2013 (avian H7N9) (Horimoto and Kawaoka, 2005; Hsieh et al., 2016). Pandemics with high mortality rates have continued to occur, and thus, novel therapeutic agents are needed (Kilbourne, 2006).

Influenza viruses belong to the Orthomyxoviridae family and contain a segmented, negative-strand RNA genome (virion RNA, vRNA). Each genome segment forms a viral ribonucleoprotein complex (vRNP) containing nucleoprotein (NP) molecules and a RNA-dependent RNA polymerase (RdRp) complex (polymerase acidic protein [PA], polymerase basic protein 1 [PB1], and PB2) (Krug and Aramini, 2009). The major surface protein, hemagglutinin (HA), binds to the sialic acid receptor on the host’s cellular surface and facilitates viral entry. The neuraminidase (NA) viral surface protein promotes the release of virions by cleaving sialic acid and also impacts viral entry (Su et al., 2009). Following viral endocytosis and fusion, the vRNPs are released into the cytoplasm by the influx of H^+^ through the M2 channel (uncoating) and then transported into the nucleus to initiate viral replication (vRNA production) and transcription (viral mRNA production) (Helenius, 1992; De Clercq and Neyts, 2007). Viral mRNA is transported to the cytoplasm and translated. Three surface proteins (HA, NA, and M2) and new vRNP are transported to the cell surface, and virion packaging and budding occur (von Itzstein, 2007).

Anti-influenza virus agents can be divided into the following classes according to their targets: M2 ion channel blockers (amantadine and rimantadine), NA inhibitors (zanamivir, oseltamivir, and peramivir), RNA polymerase inhibitors (T705 and flutimide), inosine 5′ monophosphate (IMP) dehydrogenase inhibitors (ribavirin and viramidine), interferon, and small interfering (si)RNA (De Clercq and Neyts, 2007). Although these are currently available as antiviral drugs or still being developed, there has been increasing reports of drug resistance against influenza virus due to mutations of surface proteins (Hayden and de Jong, 2011). To overcome antiviral drug resistance, molecules that could inhibit virus replication by targeting internal proteins such as polymerase, NP, and NS1 are being investigated (Patel and Kukol, 2016).

Meliae Fructus (MF) is the dried ripe fruit of *Melia toosendan* Siebold et Zuccarini, family Meliaceae. MF has been used in Chinese, Japanese, and Korean traditional medicine to treat acute or chronic inflammation, malaria, and stomach ache by roundworms as well as insecticide (Tada et al., 1999; Xie et al., 2008; Park et al., 2014). Studies have recently indicated that MF has anti-bacterial, anti-oxidant, anti-cancer, anti-inflammatory, and analgesic activities (Zhang et al., 2007; Tang et al., 2012). Although toosendanin, a compound of MF and bark of *Melia toosendan* Siebold et Zuccarini has been shown the inhibitory effect on hepatitis C virus infection by enhancing the alpha-interferon-induced signals and limonoids from MF inhibits *Mycobacterium tuberculosis* and Flavivirus (Watanabe et al., 2011; Sanna et al., 2015), the anti-influenza virus properties of an ethanolic extract of MF (EMF) has yet to be investigated. In this study, we investigated whether EMF has the anti-viral activity using an EMF pre-, co-, and post-treatment assay of Influenza A/PR/8/34 and H3N2 virus infection on Madin-Darby canine kidney (MDCK) cells in order to elucidate the inhibitory mechanism of EMF on influenza A virus infection.

## Materials and Methods

### Cells and Viruses

Madin-Darby canine kidney (ATCC CCL-34) cells were maintained in Eagle’s Minimum Essential Medium (EMEM) (Lonza, Basel, Switzerland) supplemented with 10% fetal bovine serum (FBS) and antibiotics (100 U/ml penicillin and 100 μg/ml streptomycin) at 37°C with 5% CO_2_. Influenza A viruses, Puerto Rico/8/34 (A/PR/8/34; H1N1, accession no. CY033584), Green Fluorescent Protein (GFP)-tagged A/PR/8/34 (A/PR/8/34-GFP), and KBPV-VR-32 (H3N2) were propagated in the allantoic fluid of 10-day-old chicken embryos. We received A/PR/8/34 and A/PR/8/34-GFP from Dr. Jong-Soo Lee, College of Veterinary Medicine, Chungnam National University, Republic of Korea and H3N2 were purchased from Korea Bank for Pathogenic Viruses. The virus titers were determined by using a plaque assay, as described previously (Balish et al., 2013).

### Preparation of the Ethanolic Extract of MF (EMF)

Meliae Fructus was obtained from Yeongcheon hyundai Herbal Market (Yeongcheon, Korea) and identified by Professor Ki Hwan Bae, Chungnam National University, Republic of Korea. Dried MF (30.0 g) was extracted with 390 mL 70% ethanol in a 37°C shaking incubator for 24 h. The extract solution was filtered and concentrated using a rotary vacuum evaporator (Buchi, Tokyo, Japan). Samples were freeze-dried and stored in a desiccator at 4°C before use. Sample acquisition was 5.2 g, and the yield was 17.5%.

### Cell Cytotoxicity MTS Assay

**FIGURE 1:**
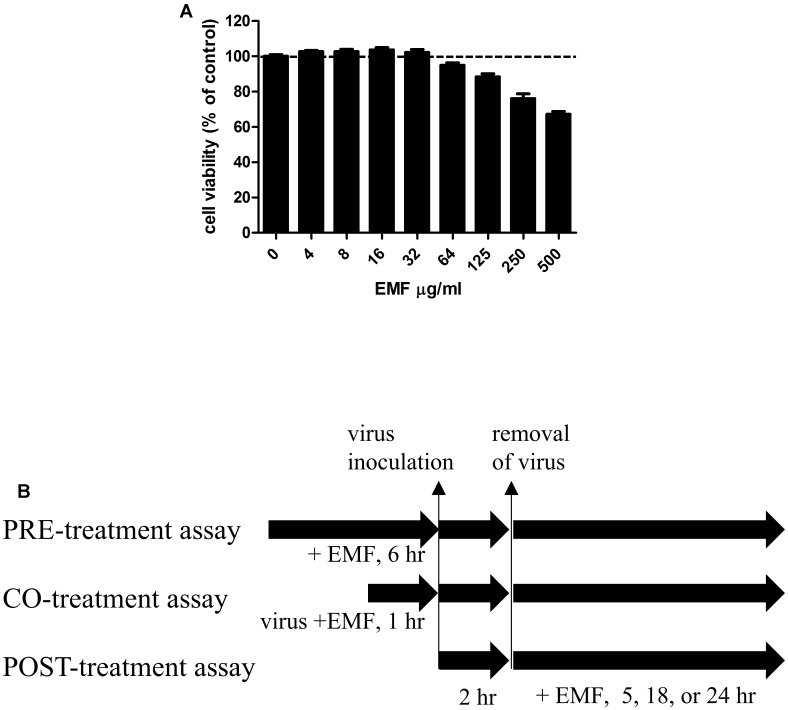
**The cytotoxicity of EMF in MDCK cells and a schematic diagram of the anti-influenza virus assay to assess the effect of EMF. (A)** To determine the cytotoxicity of EMF, the viability of MDCK cells were determined using the MTS assay after the treatment of indicated concentrations of EMF for 24 h. The data represent the mean ± SD based on three replicates in three different experiments. Statistical significance was assessed via one-way ANOVA. *P* < 0.001. **(B)** In the pre-treatment assay, the indicated concentrations of EMF were treated on MDCK cells for 6 h prior to A/PR/8/34, A/PR/8/34-GFP, or H3N2 virus (10 MOI) infection. In the co-treatment assay, 10 MOI virus and the indicated concentrations of EMF were mixed and incubated at 4°C for 1 h. MDCK cells were infected with the virus mixture for 2 h at 37°C. In the post-treatment assay, MDCK cells were infected with virus for 2 h at 37°C and were treated with the indicated concentrations of EMF for the indicated times.

Madin-Darby canine kidney cells (2 × 10^4^ cells/well) were seeded in 96-well tissue culture plates and incubated at 37°C and 5% CO_2_ overnight. EMF was added at indicated concentrations in **Figure 1A**. After 24 h incubation, 3-(4,5-dimethylthiazol-2-yl)-5-(3-carboxymethoxyphenyl)-2-(4-sulfophenyl)-2H-tetrazolium (MTS) solutions (Promega, Madison, WI, USA) were added to each well, and the cells were incubated for one more hour. The absorbance at 490 nm was recorded using the Glomax Explorer System (Promega, Madison, WI, USA). The concentration of EMF that caused the death of 50% of the cells was defined as the 50% cytotoxic concentration (CC_50_).

### Virus Infection and Antiviral Assay

Madin-Darby canine kidney cells were cultured in 96-well plates at a density of 2 × 10^4^ cells/well for 12 h. For the pre-treatment assay, before virus infection, the indicated EMF concentrations were added to the cells and incubated for 6 h. Then, EMF was removed, and the MDCK cells were washed two times with PBS. The cells were infected at 10 multiplicity of infection (MOI) using A/PR/8/34, A/PR/8/34-GFP or H3N2 at 37°C for 2 h. For the co-treatment assay, the indicated EMF concentrations were mixed with 10 MOI A/PR/8/34, A/PR/8/34-GFP or H3N2 and the mixture was incubated at 4°C for 1 h. MDCK cells (2 × 10^4^ cells/96 well) were infected with the virus mixture at 37°C for 2 h. Afterward, the virus was removed and replaced by complete EMEM containing 10 μg/mL L-1-Tosylamide-2-phenylethyl chloromethyl ketone (TPCK)-treated trypsin (Sigma, St. Louis, MO, USA). The cultures were incubated for the indicated time at 37°C and 5% CO_2_. For the post-treatment assay, the cells were infected with 10 MOI A/PR/8/34, PR/8/34-GFP or H3N2 at 37°C for 2 h. Afterward, the virus was removed, and cells were treated with indicated concentrations of EMF in complete EMEM containing 10 μg/mL TPCK-treated trypsin for the indicated time at 37°C and 5% CO_2_ (**Figure 1B**).

Viral cytopathic effect (CPE) was determined at 24 hpi by the MTS assay as described above. Viral GFP expression was observed using a fluorescence microscope (Olympus, Tokyo, Japan) and measured with a fluorescence spectrometer, Glomax Explorer System (Promega, Madison, WI, USA). The 50% effective concentration (EC_50_) values were calculated by regression analysis.

### Hemagglutination (HA) Inhibition Assay

A/PR/8/34 was diluted with PBS to achieve 4 HAU/25 μl in a round-bottomed 96-well plate and mixed with serially diluted EMF for 1 h at 4°C. Chicken red blood cells (cRBCs) (Innovative Research Inc, Southfield, MI, USA) were added to each well at a concentration of 0.5% in PBS. After incubation for 1 h at room temperature, the plate was imaged (Shoji et al., 2015).

### Neuraminidase (NA) Inhibition Assay

Neuraminidase inhibition assay was performed using the NA-Fluor^TM^ Influenza Neuraminidase Assay Kit (Applied Biosystems, Foster City, CA, USA) as per the manufacturer’s instructions. Briefly, EMF was serially diluted with assay buffer in a 96-well black plate. A/PR/8/34 in assay buffer was added to each well and incubated for 30 min at 37°C with serially diluted EMF. Each sample was mixed with 200 μM NA-Fluor Substrate in a 96-well plate. After 1 h at 37°C, the reaction was terminated by adding NA-Fluor stop solution and monitored in a fluorescence spectrometer (Promega, Madison, WI, USA) using an excitation wavelength of 365 nm and an emission wavelength range of 415–445 nm.

### Quantitative Real Time-Polymerase Chain Reaction (qRT-PCR)

Total RNA was extracted from MDCK cell lysates using an RNeasy mini kit (Qiagen, Hilden, Germany). Total RNA (500 ng) was used to synthesize cDNA using Accupower RT-PreMix (Bioneer, Daejeon, Korea). RT-qPCR was performed using AccuPower^®^ 2X Greenstar qPCR Master Mix (Bioneer, Daejeon, Korea) and a CFX96 Touch Real-Time PCR System (Bio-Rad, Hercules, CA, USA). The sense and antisense primer sequences, respectively, used are as follows: HA (5′-ttgctaaaacccggagacac-3′ and 5′-cctgacgtatttgggcact-3′); NP (5′-gaatggtgctctctgcttttga-3′ and 5′-tccactttccgtttactctcctg-3′); M2 (5′-gaaaggagggccttctacgg-3′ and 5′-tcgtcagcatccacagcac-3′); NS1 (5′-gcgatgccccattccttg-3′ and 5′-atccgctccactatctgctttc-3′); PA (5′-aagtgccataggccaggtttc-3′ and 5′-cctcatctccattccccatttc-3′); PB1 (5′-gacaacaaacaccgaaactggag-3′ and 5′-ccatcgcctccaatacacaatc-3′); PB2 (5′-ggtgcttacgggcaatcttc-3′ and 5′-tgttcgtctctcccactcactatc-3′); canine interferon (IFN)-β (5′-ccagttccagaaggaggaca-3′ and 5′-tgtcccaggtgaagttttcc-3′); canine Mx1 (5′-gaatcctgtacccaatcatgtg-3′ and 5′-taccttctcctcatattggct-3′); and canine β-actin (5′-tgccttgaagttggaaaacg-3′ and 5′-ctggggcctaatgttctcaca-3′) (Shoji et al., 2015). Relative expression was calculated by the ΔΔCt method, and β-actin expression served as an internal reference. Real-time PCR reactions were performed in triplicate.

### Immunofluorescence Staining

After 18 hpi, MDCK cells were washed twice with PBS and fixed with 10% formalin solution in TBS for 10 min. Cells were permeabilized in 0.3% Triton X-100 in TBS for 15 min and incubated with a rabbit polyclonal antibody detecting M2, PA (GeneTex, San Antonio, TX, USA), or phospho-AKT (Ser473) (Cell Signaling Technology, Danvers, MA, USA) at 4°C overnight and with a Alexa Fluor^®^ 488 goat anti-rabbit IgG antibody (Thermo Fisher Scientific, Waltham, MA, USA) at 37°C for 1 h. Nuclei were visualized by staining with DAPI (1 μg/mL). Images were captured on a fluorescence microscope (Olympus, Tokyo, Japan).

### Western Blot Analysis

Madin-Darby canine kidney cells (5 × 10^6^ cells/6-well) were harvested at the indicated time. Equal amounts of protein lysate (20 μg) in radioimmunoprecipitation assay (RIPA) buffer were separated by 12% SDS-PAGE. After transfer, polyvinylidene difluoride (PVDF) membranes were incubated at room temperature for 1 h with primary antibodies (1:1000 dilution) against phospho-AKT (Ser473) (Cell Signaling Technology, Danvers, MA, USA) and β-actin (Cell Signaling Technology, Danvers, MA, USA). Horseradish peroxidase (HRP)-conjugated secondary antibodies (1:2000 dilution) were then incubated at room temperature for 1 h. Protein bands were detected with an enhanced chemiluminescence reagent (Thermo Scientific, Waltham, MA, USA) and a ChemiDoc imaging system (Bio-Rad, Hercules, CA, USA). The relative intensities of protein bands were analyzed using Image Lab Version 5.2 densitometric analysis program (Bio-Rad, Hercules, CA, USA).

### Statistical Analysis

An unpaired Student’s *t*-test was used to assess possible significant differences (a two-tailed *p*-value) between the treatment and control groups. One-way analysis of variance (ANOVA) test was used for comparisons when more than two groups were analyzed. GraphPad Prism software version 5.0 (GraphPad Software, San Diego, CA, USA) was used. *P* < 0.05 were considered statistically significant.

## Results

### EMF CC_50_ Determination on MDCK Cells

We screened anti-influenza A virus activity of traditional herbal medicine, and we found EMF to possess potent anti-influenza A virus activity on MDCK cells. MDCK cells were widely used for the study of influenza virus, because of their high susceptibility to infection with influenza strains (Gaush and Smith, 1968).

The cytotoxicity of EMF was assessed using the MTS assay after 24 h treatment with various EMF concentrations. CC_50_ value for EMF was 531.8 ± 40.4 μg/ml (**Figure 1A**). Thus, all experiments for antiviral effects were performed at EMF concentrations below CC_50_ value of 100 μg/ml.

### EMF Suppressed Influenza A Virus Infection on MDCK Cells in Pre- and Co-treatment Assay

**FIGURE 2:**
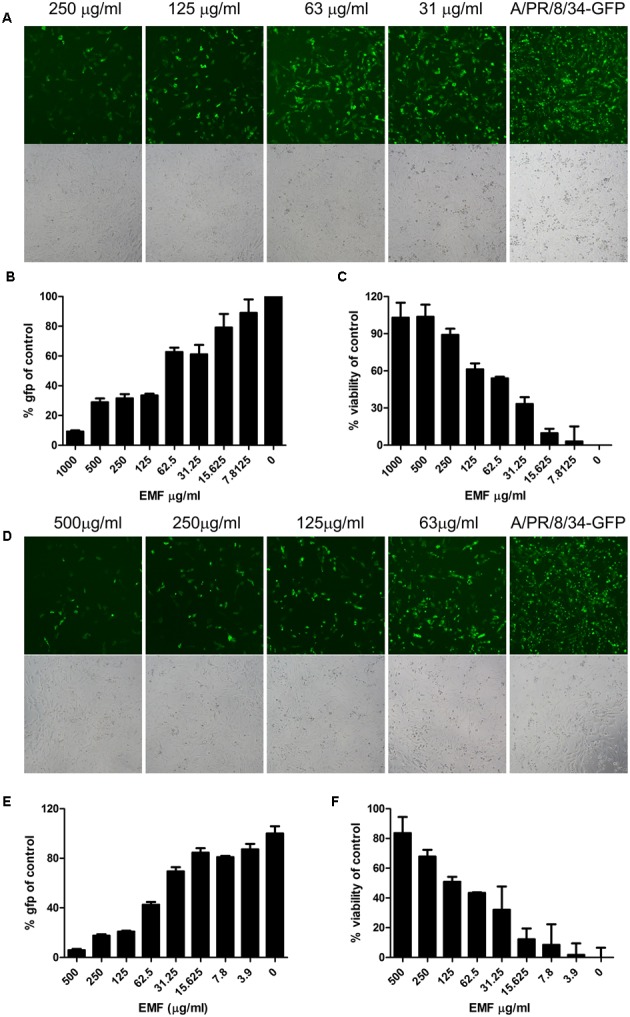
**EMF inhibited influenza virus infection and cytopathic effect in a dose-dependent manner in pre- and co-treatment assays. (A–C)** In the pre-treatment assay, MDCK cells were pre-treated with serially diluted concentrations of EMF for 6 h prior to infection with the A/PR/8/34-GFP virus (10 MOI). **(D–F)** In the co-treatment assay, the serially diluted concentrations of EMF were mixed with 10 MOI A/PR/8/34-GFP and incubated at 4°C for 1 h. The MDCK cells were infected with the virus mixture at 37°C for 2 h. Virus-expressed GFP protein was detected by fluorescence microscopy (upper panel, fluorescence; bottom, phase contrast microscopy) at 20× magnification **(A,D)** and by fluorescence spectrometry **(B,E)** at 24 hpi. Virus-induced CPE in MDCK cells was measured using the MTS assay at 24 hpi **(C,F)**. The data represent the mean ± SD based on three replicates. All data are representative of three independent experiments. Statistical significance was assessed via one-way ANOVA. *P* < 0.001.

To evaluate anti-influenza A virus activity, we assessed virus-induced CPE and used a modified GFP assay as previously described (Talactac et al., 2015). MDCK cells were infected with A/PR8/34-GFP (10 MOI) for 2 h after 6 h pre-treatment with the serially diluted concentration of EMF in the pre-treatment assay. After 24 hpi, EMF treated MDCK cells significantly reduced GFP expression in a dose dependent manner (**Figures 2A,B**). Consistent with these data, MTS assay data showed that pre-treatment of EMF can protect MDCK cells from A/PR8/34 and H3N2 virus-induced cell death in a dose-dependent manner (**Figure 2C** and Supplementary Figure 2A). These data suggested that EMF inhibited the A/PR8/34-virus-induced CPE and GFP expression with an EC_50_ of 68.2 ± 6.4 μg/ml.

In the co-treatment assay, serially diluted EMF concentrations were mixed with A/PR8/34-GFP and incubated at 4°C for 1 h, and then MDCK cells were inoculated with the mixture for 2 h. After 24 hpi, we found that EMF had inhibitory activity against A/PR/8/34-GFP in a dose-dependent manner with an EC_50_ value of 48.9 ± 13.4 μg/ml as determined by GFP expression (**Figures 2D,E**) and CPE analysis (**Figure 2F**). It could inhibit H3N2 virus-induced CPE in a dose-dependent manner (Supplementary Figure 2B). However, in the post-treatment assay, EMF showed no inhibitory effect (Supplementary Figure 1). Therefore, these data indicated that EMF has anti-influenza A virus activity in the pre- and co-treatment assays, in a dose-dependent manner, but not in post-treatment assay.

### EMF Has HA and NA Inhibition Activity

**FIGURE 3:**
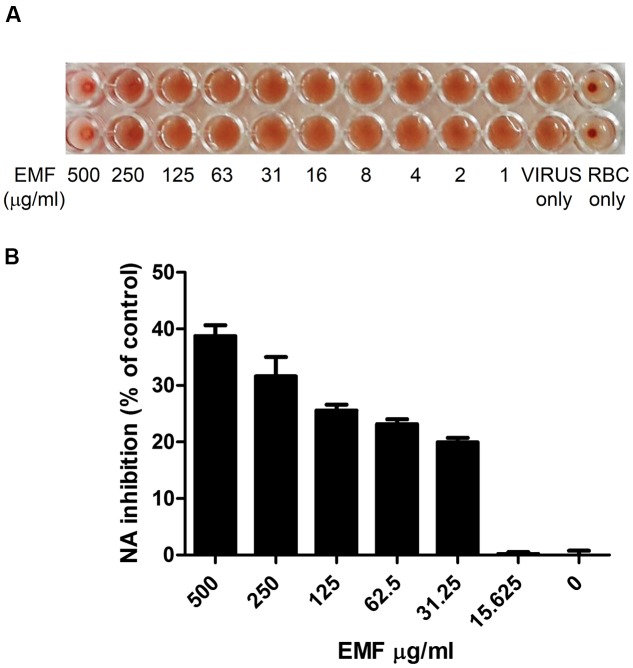
**EMF inhibited HA and NA activities of A/PR/8/34. (A)** A/PR/8/34 virus was diluted with PBS at 4 HAU, mixed with serially diluted concentrations of EMF for 1 h at 4°C in duplicates. 0.5% cRBCs in PBS were added to each well and incubated for 1 h at room temperature and imaged. **(B)** A/PR/8/34 was incubated with serially diluted EMF for 30 min at 37°C, and 200 μM NA-Fluor substrate was added. After 1 h incubation at 37°C, the reaction was terminated by adding NA-Fluor stop solution and monitored via fluorescence spectrometry. Data are presented as the mean ± SD based on three replicates in three independent experiments. Statistical significance was assessed via one-way ANOVA. *P* < 0.001.

The co-treatment assay results indicated that EMF directly affected influenza virus. Therefore, we evaluated whether EMF inhibits hemagglutination (HA) activity. The HA inhibition assay showed that EMF inhibited viral attachment onto cRBCs at 500 μg/ml (**Figure 3A**). This result indicated that EMF inhibits HA activity. Next, we conducted an NA inhibition assay to determine whether NA activity was impaired by EMF. NA activity of A/PR/8/34 was inhibited in a dose-dependent manner upon EMF treatment with an EC_50_ value of 428.2 ± 11.6 μg/ml (**Figure 3B**), implying that EMF affects NA activity. Collectively, these data strongly suggested that EMF inhibits HA and NA activity of influenza virus, A/PR/8/34.

### EMF Impaired Viral mRNA Synthesis

**FIGURE 4:**
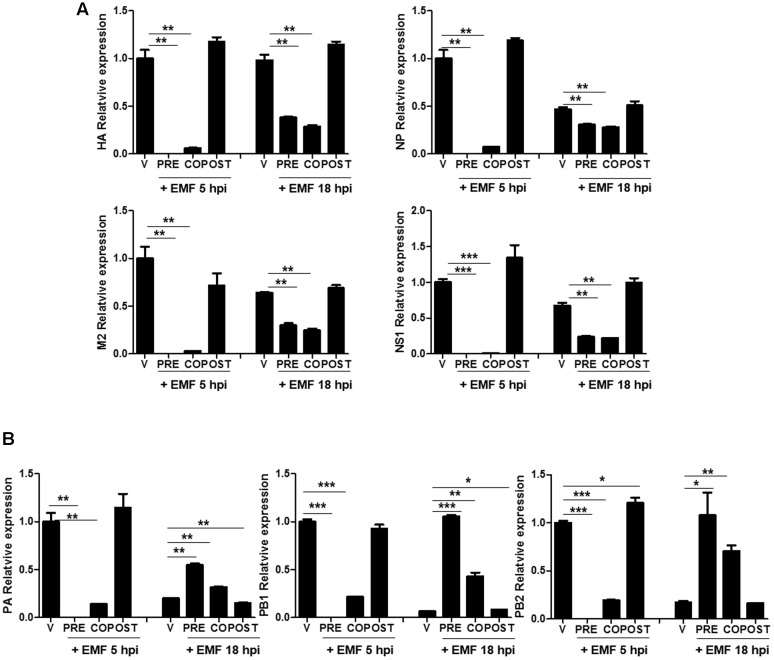
**EMF affected the level of A/PR/8/34 mRNA synthesis in pre- and co-treatment assays**. In pre (PRE)-, co (CO)-, and post (POST)-treatment assays with EMF at 100 μg/ml, A/PR/8/34-infected MDCK cells at 10 MOI were analyzed the relative levels of viral HA, NP, M2, and NS1 mRNA **(A)** and PA, PB1, and PB2 mRNA **(B)** at 5 and 18 hpi via qRT-PCR, normalized to β-actin mRNA. Data are presented as means ± SD based on three replicates and are representative of three independent experiments. n. d., not detected. V, infected control MDCK cells. Statistical significance was assessed via the Student’s *t*-test. ^∗^*P* < 0.05; ^∗∗^*P* < 0.01; ^∗∗∗^*P* < 0.001.

We further investigated whether EMF inhibits viral mRNA synthesis. After pre-, co-, and post- EMF treatment, at a concentration of 100 μg/ml, A/PR/8/34-infected MDCK cells were harvested and relative mRNA expression level of viral genes at 5 and 18 hpi were assessed by qRT-PCR. HA, NP, M2, and NS1 mRNA synthesis was suppressed in pre- and co-treated MDCK cells with EMF at 5 and 18 hpi (**Figure 4A**). Inhibition of mRNA synthesis was more robust at 5 hpi than at 18 hpi. In post-treated cells with EMF, EMF did not affect HA, NP, M2, and NS1 mRNA synthesis, consistent with previous data (Supplementary Figure 1).

Moreover, three subunits of the RdRp complex (PA, PB1, and PB2) mRNA synthesis were decreased in pre- and co-treated cells with EMF at 5 hpi, but this was not observed in post-treated cells (**Figure 4B**). However, PA, PB1, and PB2 mRNA synthesis were more increased in pre- and co-treated cells with EMF at 18 hpi compared with virus infected and post-EMF treated cells. Our findings suggested that pre- and co-treatment with EMF inhibits viral mRNA synthesis of HA, NP, M2, NS1, PA, PB1, and PB2 at 5 hpi, however PA, PB1, and PB2 mRNA synthesis is enhanced in pre- and co-treated cells with EMF compared with infected control and EMF post-treated cells at 18 hpi.

### EMF Reduced M2 Protein Levels and Affected PA Protein Localization

**FIGURE 5:**
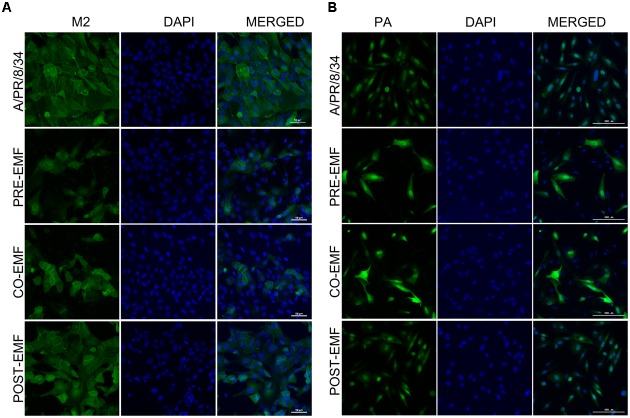
**EMF reduced the M2 protein and localized the PA protein in the nuclei and cytoplasm of infected MDCK cells in pre- and co-treatment assays**. In pre (PRE-EMF)-, co (CO-EMF)-, and post (POST-EMF)-treatment assays with EMF at 100 μg/ml, A/PR/8/34-infected MDCK cells at 10 MOI were fixed at 18 hpi. The green foci indicated the presence of M2 **(A)** or PA **(B)** viral proteins (left column), and nuclei were stained with DAPI (middle column). The viral protein and DAPI images were merged (right column). Data are representative of three independent experiments. A/PR/8/34, virus-infected control MDCK cells. Scale bars, 50 μm **(A)** and 100 μm **(B)** at 40× magnification.

To confirm the expression of A/PR/8/34 and H3N2 viral protein expression in pre-, co-, and post- treated cells with EMF at 100 μg/ml, we performed immunofluorescence analysis after 18 hpi with A/PR/8/34 and H3N2. Viral M2 protein expression was suppressed in EMF pre- and co-treated cells after 18 hpi compared with infected control and EMF post-treated cells, which is consistent with observed M2 mRNA levels (**Figure 5A** and Supplementary Figure 2C).

We also examined the expression of PA protein in pre-, co-, and post-treatment assay with EMF at 18 hpi. PA proteins were localized in the nuclei of infected MDCK cells at 18 hpi, as previously demonstrated (**Figure 5B** and Supplementary Figure 2D) (Jones et al., 1986). However, PA protein expression was distributed in the nucleus and cytoplasm of EMF pre-and co-treated MDCK cells, whereas PA proteins in post- EMF treated MDCK cells were still localized in the nucleus.

While M2 protein expression decreased, PA proteins accumulated and localized in not only the nucleus but also cytoplasm in influenza-infected cells upon pre- and co-treatment with EMF at18 hpi. However, there were no differences between infected post-treated cells with EMF and infected control MDCK cells.

### EMF Inhibited PI3K/AKT Phosphorylation Induced by Influenza A Virus Infection

**FIGURE 6:**
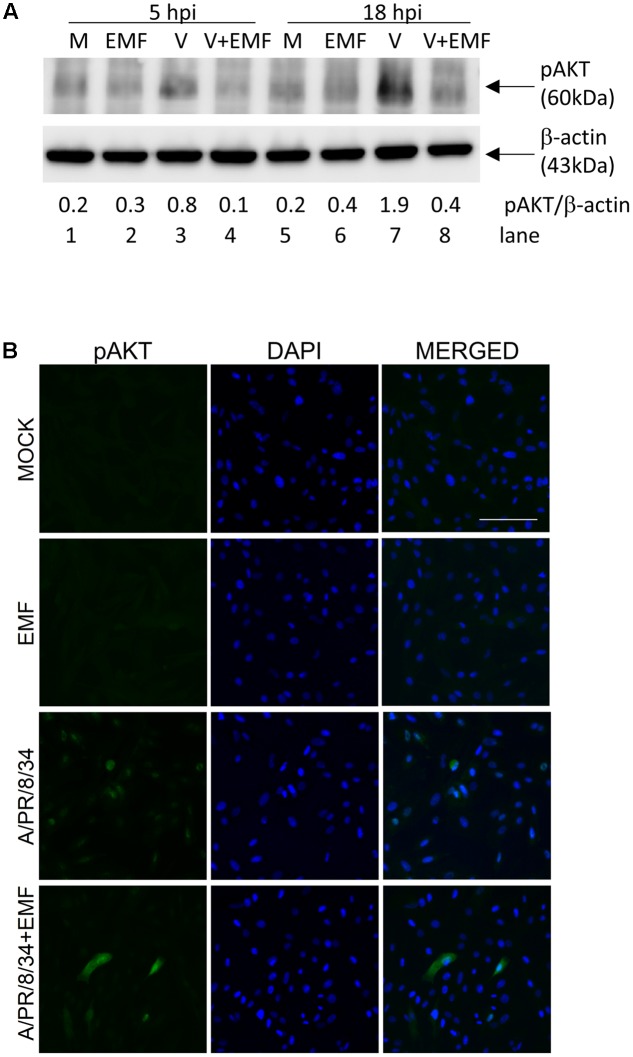
**EMF inhibited virus-induced phosphorylation of AKT**. MDCK cells were treated with EMF at 100 μg/ml for 6 h prior to A/PR/8/34 infection at 10 MOI. **(A)** MDCK cell lysates were harvested at 5 and 18 hpi, and phosphorylated AKT protein were detected by Western blot, with β-actin serving as an internal control. The relative ratio of pAKT/β-actin protein bands was calculated. M, untreated cells. EMF, EMF-treated cells. V, virus-infected cells. V + EMF, EMF pre-treated cells prior to virus infection. **(B)** Cells were fixed at 18 hpi and stained with the anti-pAKT antibody (left column, green) and DAPI for nucleus (middle column, blue). The viral protein and DAPI images were merged (right column). Data are representative of three independent experiments. Scale bars, 100 μm.

Only pre- and co-treatment with EMF suppressed influenza virus infection, which suggested that EMF affects early events in infection. It is known that PI3K (phosphatidylinositol 3-kinase)/AKT (a serine/threonine kinase) phosphorylation occurs during viral entry (Ehrhardt et al., 2006), so we examined whether EMF inhibits AKT phosphorylation after A/PR/8/34 infection. After pre-treatment of EMF at 100 μg/ml for 6 h, MDCK cells were infected with A/PR/8/34, and Western blot analysis was performed to detect phosphorylated AKT at 5 and 18 hpi (**Figure 6A**). Influenza virus infection induced increased AKT phosphorylation (lanes 3 and 7) at 5 and 18 hpi as previously reported. However, virus-induced phosphorylation was inhibited by pre-treatment with EMF at 5 and 18 hpi (lanes 4 and 8).

We also used immunofluorescence analysis to confirm this data (**Figure 6B**). In pre-EMF treated cells, the number of cells expressing AKT phosphorylation decreased compared with control cells at 18 hpi. Therefore, our data confirmed that pre-EMF treatment decreased phospho-AKT expression induced by viral infection.

### Pre- and Co-treatment with EMF Increased Antiviral Factor Mx1 at 18 hpi

**FIGURE 7:**
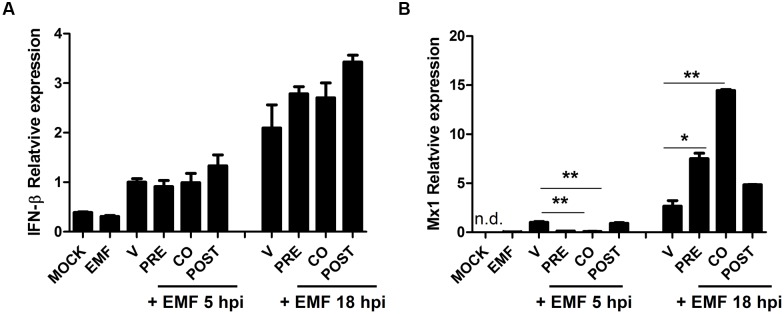
**EMF up-regulated antiviral protein Mx1 in pre- and co- treatment assays**. In pre (PRE)-, co (CO)-, and post (POST)-treatment assays with EMF at 100 μg/ml, A/PR/8/34-infected-MDCK cells at 10 MOI were analyzed for the relative mRNA level of antiviral factors, IFN-β **(A)** and Mx1 **(B)** at 5 and 18 hpi using qRT-PCR, normalized to β-actin mRNA. Data are presented as mean ± SD based on three replicates and are representative of three independent experiments. MOCK, untreated cells. EMF, 100 μg/ml EMF treated cells. V, infected control MDCK cells. n. d., not detected. Statistical significance was assessed via the Student’s *t*-test. ^∗^*P* < 0.05; ^∗∗^*P* < 0.01.

We examined whether EMF treatment affects the host cell immune response induced by A/PR/8/34. Particularly, we analyzed the level of antiviral factors, IFN-β and Mx1 mRNA in A/PR/8/34 infected MDCK cells upon pre-, co-, and post-treatment of EMF at 100 μg/ml at 5 and 18 hpi via qRT-PCR. IFN-β mRNA levels were not different between pre-, co-, and post-EMF treatment and control MDCK cells at 5 and 18 hpi (**Figure 7A**). However, Mx1 mRNA levels were significantly down-regulated in pre- and co-treated MDCK cells at 5 hpi, but Mx1 was up-regulated in pre- and co- EMF treated MDCK cells at 18 hpi compared with control and post-EMF treated MDCK cells (**Figure 7B**). Therefore, up-regulation of Mx1 by EMF may contribute to inhibiting influenza virus infection in pre- and co-EMF treated MDCK cells at 18 hpi.

## Discussion

While MF is known for its anti-bacterial, anti-oxidant, anti-cancer, anti-inflammatory, and analgesic activities, anti-influenza virus activity of MF has yet to be reported. In this study, we found that ethanolic extract of MF demonstrates potent anti-influenza A virus activity in MDCK cells.

Viral replication was only inhibited in pre- and co- EMF treated MDCK cells, and not in post-treated cells, at 24 hpi (**Figure 2** and Supplementary Figure 2). These findings suggested that EMF functions before viral adsorption or during infection and does not affect post-virus infection.

The surface HA protein of influenza virus binds to the sialic acid receptor on the host’s cell surface and mediates viral entry. NA is known to promote the release and spread of progeny virions and can also enhance HA-mediated viral membrane fusion and virion infectivity in early stages of influenza virus replication (Matrosovich et al., 2004; Su et al., 2009). We found EMF inhibited HA and NA activity (**Figure 3**), implying that the inhibition of virus infection by EMF results from blocking the HA-sialic acid receptor interaction and NA activity in the early stages of infection. The PI3K/AKT signaling pathway regulates viral entry, and the inhibition of AKT kinase activity suppresses viral entry and replication (Ehrhardt et al., 2006; Hirata et al., 2014). We found that EMF treatment decreased AKT phosphorylation (**Figure 6**). Thus, EMF treatment interferes with an early process of virus uptake. It has previously been shown that limonoid compounds from MF inhibit West Nile virus infection only when added during virus infection, suggesting that the inhibition occurs at the entry or an early stages of life cycle (Sanna et al., 2015).

Ethanolic extract of MF treatment also affected viral RNA polymerase including PA, PB1, and PB2 mRNA synthesis and PA protein localization. EMF inhibited viral HA, NP, M2, NS1, PA, PB1, and PB2 viral mRNA synthesis at 5 hpi, but PA, PB1, and PB2 mRNA synthesis was higher in EMF pre- and co-treated cells compared with virus-infected and EMF post-treated cells at 18 hpi (**Figure 4**). Moreover, M2 protein expression was decreased upon pre- and co-treatment with EMF, but PA proteins were accumulated and localized in not only the nucleus but also the cytoplasm of infected cells at 18 hpi upon pre- and co- EMF treatment (**Figure 5** and Supplementary Figure 2B).

Polymerase basic proteins 1 are essential for viral RNA polymerase activity, PB2 proteins recognize and bind to host 5′ mRNA cap structure involved in transcription initiation, and PA proteins possess endonucleolytic activity for the viral cap-snatching process (Rodriguez-Frandsen et al., 2015). PA, PB1, and PB2 proteins are predominantly associated with the nuclei of influenza virus infected cells (Jones et al., 1986). It has been reported that PA proteins induce the proteolytic process to decrease its own accumulation, and the nuclear transport of PA proteins could be a prerequisite for proteolysis (Sanz-Ezquerro et al., 1995, 1996). Moreover, ability of PA proteins-mediated induction of proteolysis is linked to the replication activity of the polymerase (Perales et al., 2000). Therefore, our data suggested that EMF impaired the proteolytic ability, the nuclear transport and the polymerase activity of PA proteins, resulting in accumulation of PA proteins in the cytoplasm of influenza virus infected cells and inhibition of virus replication. Because PA proteins of the RNA polymerase complex are promising targets for anti-influenza virus agents, elucidating the mechanism of impaired PA function by EMF is critical.

Pre-and co-treatment with EMF increased the antiviral protein Mx1 at 18 hpi in spite of the lower level of Mx1 at 5 hpi. Mx1 proteins are important antiviral factors against RNA viruses including influenza viruses at an early stage in their life cycle (Haller et al., 2007). Mx1 inhibits influenza virus by disrupting the PB2–NP protein interaction, interfering with ribonucleoprotein complex assembly, and decreasing viral polymerase activity (Verhelst et al., 2012). Thus, up-regulation of Mx1 by EMF may play an important role in inhibiting infection in EMF pre-and co- treated cells. Interestingly, Mx1 gene expression is induced by type I and III IFN, but not directly by viruses (Haller et al., 2015). However, EMF treatment affected the level of Mx1 mRNA, not IFN-β. Thus, it will be interesting to find the Mx1-inducing factor and the mechanism of upregulation upon EMF treatment.

In summary, our study suggested that an ethanolic extract of Meliae Fructus inhibited influenza A virus infection by affecting viral entry, PA proteins of the RNA polymerase complex, and Mx1 induction. Further investigation is needed to characterize active compounds and to elucidate their specific mechanism against influenza A virus. It may be helpful to develop potential candidates for novel anti-influenza agent.

## Author Contributions

Conceived and designed the experiment: JYM, W-KC, and Y-HJ. Performed experiments: Y-HJ. Analyzed the data: J-GC and Y-HJ. Wrote the paper: Y-HJ.

## Conflict of Interest Statement

The authors declare that the research was conducted in the absence of any commercial or financial relationships that could be construed as a potential conflict of interest.
