# Supplementary figures and images for: Ethanolic Extract of Melia Fructus Has Anti-influenza A Virus Activity by Affecting Viral Entry and Viral RNA Polymerase
Source: Front Microbiol. 2017 Mar 28;8:476. doi: 10.3389/fmicb.2017.00476 (PMC5368190; doi:10.3389/fmicb.2017.00476)

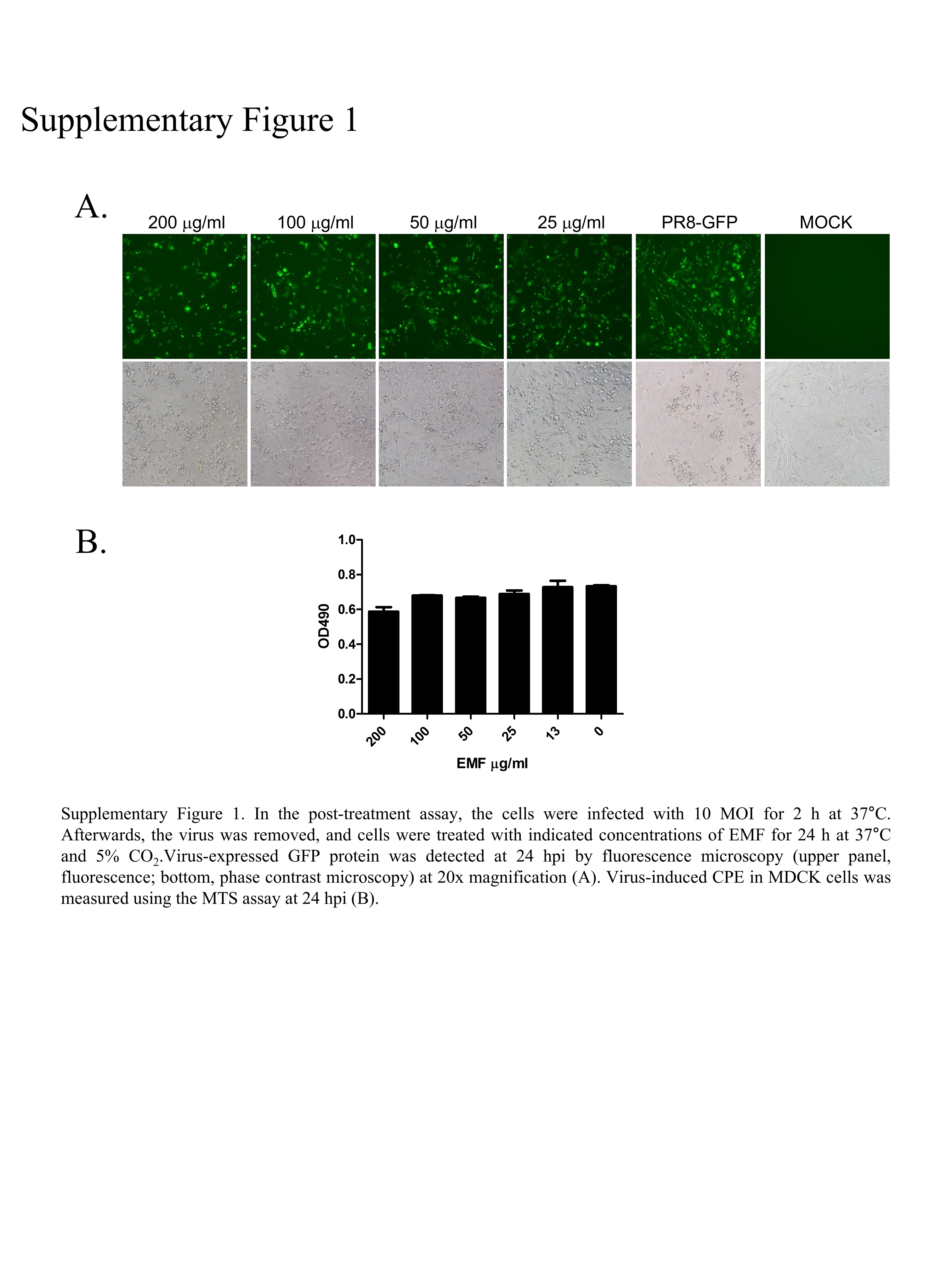

Supplement: Supplementary file 1 [file Image_1.tif]

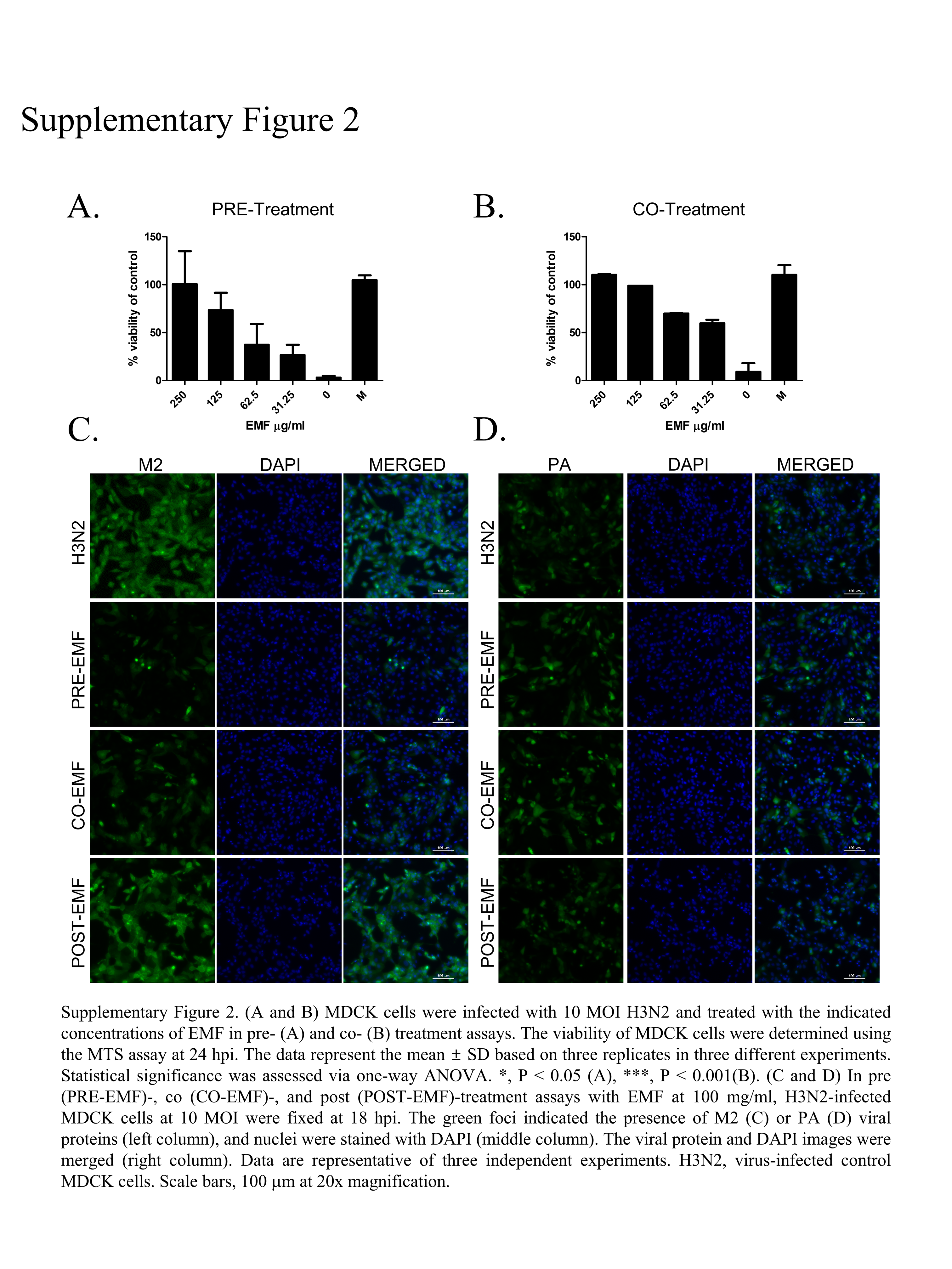

Supplement: Supplementary file 2 [file Image_2.tif]
